# Proinflammatory intervertebral disc cell and organ culture models induced by tumor necrosis factor alpha

**DOI:** 10.1002/jsp2.1104

**Published:** 2020-06-19

**Authors:** Jie Du, Judith‐J. Pfannkuche, Gernot Lang, Sonja Häckel, Laura B. Creemers, Mauro Alini, Sibylle Grad, Zhen Li

**Affiliations:** ^1^ AO Research Institute Davos Davos Switzerland; ^2^ Department of Orthopedics University Medical Center Utrecht Utrecht The Netherlands; ^3^ Department of Orthopedics and Trauma Surgery Medical Centre—Albert‐Ludwigs‐University of Freiburg, Faculty of Medicine, Albert‐Ludwigs‐University of Freiburg Freiburg Germany; ^4^ Department of Orthopaedic Surgery and Traumatology, Inselspital Bern University Hospital, University of Bern Bern Switzerland

**Keywords:** 3R, cytokines, inflammation, intervertebral disc, regeneration, spine

## Abstract

Inflammation plays an important role in the pathogenesis of intervertebral disc (IVD) degeneration. The proinflammatory cytokine tumor necrosis factor alpha (TNF‐α) has shown markedly higher expression in degenerated human disc tissue compared with healthy controls. Anti‐inflammatory treatment targeting TNF‐α has shown to alleviate discogenic pain in patients with low back pain. Therefore, in vitro and ex vivo inflammatory models utilizing TNF‐α provide relevant experimental conditions for drug development in disc degeneration research.

The current method article addressed several specific questions related to the model establishment.

(a) The effects of bovine and human recombinant TNF‐α on bovine nucleus pulposus (NP) cells were compared. (b) The required dose for an inflammatory IVD organ culture model with intradiscal TNF‐α injection was studied. (c) The effect of TNF‐α blocking at different stages of inflammation was evaluated.

Outcomes revealed that bovine and human recombinant TNF‐α induced equivalent inflammatory effects in bovine NP cells. A bovine whole IVD inflammatory model was established by intradiscal injection of 100 ng TNF‐α/ cm^3^ disc volume, as indicated by increased nitric oxide, glycosaminoglycan, interleukin 6 (IL‐6), and interleukin 8 (IL‐8) release in culture media, and upregulation of MMP3, ADAMTS4, IL‐8, IL‐6, and cyclooxygenase (COX)‐2 expression in NP tissue. However, results in human NP cells showed that the time point of anti‐inflammatory treatment was crucial to achieve significant effects. Furthermore, anticatabolic therapy in conjunction with TNF‐α inhibition would be required to slow down the pathologic cascade of disc degeneration.

## Graphical abstract

This article describes the methods for establishment of proinflammatory intervertebral disc cell and organ culture models induced by TNF‐a.

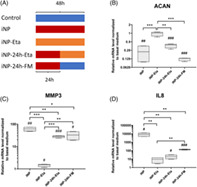

## 1 INTRODUCTION

Low back pain (LBP) is the leading cause of disability worldwide.^1^ One major cause for chronic LBP is symptomatic intervertebral disc degeneration (IVDD).^2^, ^3^, ^4^ IVDD is characterized by extracellular matrix (ECM) degradation, accelerated cartilaginous, and bone remodeling, release of proinflammatory cytokines, altered spine biomechanics, angiogenesis and neoinnervation, altogether potentially leading to chronic LBP, and disability.^5^, ^6^, ^7^, ^8^, ^9^ IVDD can be induced by mechanical stress, trauma, infection, genetic predisposition, and inflammation.^10^, ^11^, ^12^, ^13^, ^14^, ^15^, ^16^

Inflammation plays a major role in disc degeneration, as proinflammatory cytokines (ie, tumor necrosis factor alpha [TNF‐α], interleukin 1 beta [IL‐1β], interleukin 6 [IL‐6], interleukin 8 [IL‐8], interleukin 17 [IL‐17], and interferon gamma [IFN‐γ]) induce and trigger discal ECM breakdown and accelerated catabolism by stimulation of catabolic enzymes such as matrix metalloproteinases (MMPs) and a disintegrin and metalloproteinase with thrombospondin motifs (ADAMTS).^6^, ^17^, ^18^, ^19^, ^20^ Proinflammatory cytokines have shown elevated expression in degenerative and symptomatic compared to healthy and asymptomatic IVDs.^16^, ^21^ Since therapeutic approaches for IVDD remain limited, biological anti‐inflammatory approaches to IVD regeneration have gained increasing interest. In cases of refractory LBP due to IVDD, anti‐inflammatory and/or anti‐degenerative therapies such as cytokine inhibition may relieve pain and slow down the progression of the disease.^22^, ^23^, ^24^, ^25^, ^26^, ^27^, ^28^, ^29^, ^30^, ^31^ Several studies indicated that cyclooxygenase‐2 (COX2) inhibitors can reduce the inflammatory response in different models.^25^, ^26^, ^27^ Soluble TNF receptor type II is able to significantly attenuate the effects of TNF‐α on primary human IVD cells in vitro.^28^ Intradiscal administration of a TNF‐α inhibitor, Etanercept, in LBP patient can alleviate intractable discogenic LBP for up to 4 weeks.^31^

A degenerative disc exhibits increased TNF‐α expression, not only produced by immunocytes, but also by disc cells themselves.^15^, ^19^, ^32^ Furthermore, TNF‐α can induce nucleus pulposus (NP) cells to produce other cytokines and chemokines that can further enhance the inflammatory state by recruiting and activating immune cells.^33^ So far, it is widely accepted that TNF‐α contributes to disc degeneration by decreasing the anabolism and increasing the catabolism of ECM.^34^ Additionally, exogenous TNF‐α induces neuropathology and sensory nerve growth into IVD, which indicated TNF‐α might be the chemical mediator of discogenic pain.^35^, ^36^ Therefore, multiple in vitro, ex vivo, and in vivo inflammatory IVD models have been established with TNF‐α.^20^, ^28^, ^37^, ^38^, ^39^ NP cells cultured with TNF‐α in vitro showed upregulated expression of catabolic enzymes, ADAMTS 4&5 and MMP‐1,‐2,‐3,‐13, and inflammatory mediators, IL‐1β, IL‐6, IL‐8, and COX2, downregulated expression of ECM markers collagen II, aggrecan, and versican.^34^, ^40^, ^41^, ^42^, ^43^, ^44^ TNF‐α has been shown to induce MMP3 expression via nuclear factor κB (NF‐κB) and mitogen‐activated protein kinase pathways.^45^ Intradiscal injection of TNF‐α in a porcine model was sufficient to induce early‐stage disc degeneration, characterized by matrix loss, annular fissure formation, and vascularization.^46^ Lai et al reported that annular puncture with TNF‐α injection enhanced painful behavior with disc degeneration in a rat model.^39^

Ex vivo explant culture models bridge the gap between in vitro and in vivo systems and reveal many advantages by maintaining the native tissue environment and decreasing the consumption of experimental animals. Compared with the small animals like mouse, rat and rabbit, the IVDs from large animals such as sheep, dog and cow are more similar to human. They show comparable size and loss of notochordal cells in early adulthood as human IVD.^47^, ^48^ Notochordal cells have been reported to present anti‐inflammation and regenerative effect in IVDs.^49^, ^50^ With those similarities, many bovine caudal IVD organ culture models were established. van Dijk et al developed a NP tissue explant culture model, and found that using polyethylene glycol to raise culture medium osmolarity was able to maintain the NP tissue specific matrix composition.^25^, ^51^ Whole bovine caudal IVD cultured under either limited glucose condition or high‐frequency loading condition led to a significant drop in cell viability, while combined treatment with limited glucose and high‐frequency loading resulted in an additive increase in cell death in both the NP and annulus fibrosus (AF), and an increase in MMP13 gene expression.^52^ Purmessur et al cultured whole IVD organ excluding the endplates with exogenous TNF‐α in medium. Aggrecan degradation products and β‐galactosidase staining were enhanced by TNF‐α on day 21 without any recovery, when TNF‐α was removed on day 7.^38^ Recently, our group has developed a proinflammatory and degenerative IVD whole organ culture system to investigate the proinflammatory and degenerative microenvironment operant in IVDD. Results indicated that a combination of detrimental dynamic loading, nutrient deficiency and intradiscal TNF‐α injection could synergistically simulate the proinflammatory and degenerative disease condition. However, intradiscal TNF‐α injection alone did not lead to a significant inflammatory effect.^7^

In the present study, we sought to establish TNF‐α induced in vitro and ex vivo IVD inflammation models, which would represent preclinical testing systems for screening of anti‐inflammatory drugs for disc degeneration treatment. Specifically, the following questions were addressed within this study:

1. Does TNF‐α from bovine and human have the same proinflammatory effect on bovine NP cells?
2. What is the optimal dose of TNF‐α when utilized within an IVD inflammation organ culture model induced by TNF‐α intradiscal injection?
3. Does TNF‐α inhibition at different stages of inflammation have equal anti‐inflammatory and/or regenerative effects on NP cells?

## 2 MATERIALS AND METHODS

### 2.1 Medium selection

Alpha Minimum Essential Medium (αMEM) has shown an advantage compared with Dulbecco's Minimum Essential Medium (DMEM) in terms of numbers and quality of cells acquired in mesenchymal stem cells isolation and expansion.^53^ In the current study, human and bovine NP cells isolation and expansion were performed with αMEM according to previous publication.^54^ DMEM contains much higher amount of vitamins, amino acids and glucose than αMEM. Therefore, cells and IVD organ culture experiments with TNF‐α and Etanercept were performed with DMEM, due to a much higher cell density and nutrition requirement in these experiments.

### 2.2 NP cells isolation and expansion

Human NP cells were isolated from traumatic IVDs (2 donors, 34/49 years old, male) with ethical approval (Cantonal Ethic Commission Bern 2016). General consent was obtained from all patients before surgery. All studies were performed in accordance with the ethical standards as laid down in the 1964 Declaration of Helsinki and its later amendments or comparable ethical standards. The IVDs were classified as mildly degenerated by MRI (Pfirrmann grade 2‐3). Bovine NP cells were isolated from caudal intervertebral discs of 6 to 12‐month‐old calves from local abattoirs immediately after death. NP cell isolation was performed as described previously.^55^ The collected NP tissue was cut into small pieces. Human NP tissue was incubated with red blood cell lysis buffer (155 mM NH_4_Cl, 10 M KHCO_3_, and 0.1 mM EDTA in Milli‐Q water) to remove the red blood cells. The chopped tissue was digested with 0.2% w/v Pronase (Roche, Mannheim, DE) in αMEM (Gibco, Paisley, UK) for 1 hr, then digested with 65 U/mL collagenase type II (Worthington, Lakewood, NL) in αMEM / 10% fetal bovine serum (FBS, PAN Biotech, Germany) in a spinner flask for 12 to 14 hours at 37°C. The digested cell suspension was filtered through a 100 μm cell strainer to obtain a single‐cell suspension. NP cells were expanded in αMEM supplemented with 10% FBS and 100 U/mL penicillin and 100 mg/mL streptomycin (1% P/S, Gibco, Paisley, UK), incubated at a hypoxic condition of 2% O_2_ at 37°C. Culture medium was changed twice a week. Passage 2 and 3 NP cells were used in the current study.

### 2.3 Effect of human and bovine recombinant TNF‐α on bovine NP cells

Bovine NP cells were seeded at a concentration of 60 000/cm^2^ in 12‐well plates with DMEM medium (containing 4.5 g/L glucose) supplemented with 10% FBS. After cell attachment (24 hours after cell seeding), the medium was exchanged to serum‐free experimental medium (DMEM supplemented with 1% ITS+, 1% nonessential amino acid [NEAA, Gibco, Paisley, UK], 50 μg/mL ascorbate 2 phosphate and 1% P/S) with or without inflammatory inducers 10 ng/mL human recombinant TNF‐α (R&D systems, Zug, Switzerland) or 10 ng/mL bovine recombinant TNF‐α (R&D Systems, Zug, Switzerland). After another 72 hours of culture, the cell monolayer was lysed and RNA was isolated for gene expression analysis.

### 2.4 Effect of human recombinant TNF‐α and TNF‐α inhibition on human NP cells

Human NP cells were seeded into a six well‐plate at a cell density of 30 000/cm^2^. One day after seeding, cells were treated with 10 ng/mL (low dose) or 50 ng/mL (high dose) TNF‐α in serum‐free experimental medium as described above for bovine NP cells TNF‐α experiments. The samples were collected at three timepoints, 6, 24, and 48 hours after treatment, for gene expression analysis.

To investigate the effect of TNF‐α blocking with the TNF‐α inhibitor Etanercept (Enbrel, Pfizer, New York, New York), NP cells were seeded as described above and cultured for 24 hours to allow for cell attachment. Hereafter, cells were divided into 4 different groups: (1) iNP—cells were treated with 10 ng/mL TNF‐α for 48 hours, (2) iNP‐Eta—cells were treated with 10 ng/mL TNF‐α and immediately after 1 μg/mL Etanercept was added for 48 hours, (3) iNP‐24 hours‐Eta—cells were treated with 10 ng/mL TNF‐α, 24 hours after 1 μg/mL Etanercept was added, and (4) iNP‐24 hours‐FM—cells were treated with 10 ng/mL TNF‐α, 24 hours after replaced to fresh medium without TNF‐α. Cells treated with serum‐free culture medium as described above served as negative control. All the cells were harvested for gene expression analysis at 72 hours after seeding. The concentration of Etanercept used here was selected according to previous studies, showing that Etanercept at 0.01, 0.1 and 1 μg/mL induced less than 8% cell death in TNF‐α transfected Jurkat cells, and in human NP cells and AF cells cultured with Etanercept at 100, 250, 500, 1000, and 2000 μg/mL, cell proliferation was only suppressed with Etanercept at 500 μg/mL or higher.^56^, ^57^ Therefore, the selected Etanercept concentration at 1 μg/mL was assumed to have no cytotoxic effect on NP cell culture in vitro.

### 2.5 IVDs dissection

Bovine caudal IVDs were collected from fresh sacrificed 6 to 12‐month‐old calves from local slaughterhouses. Disc dissection was performed as described previously.^58^ Briefly, most of the muscle and soft tissue were removed, whole IVDs with cartilage endplates (EPs) were isolated with a band saw and redundant vertebral bone and growth plate were carefully cut off to ensure two parallel planes of discs. Disc height and diameter was then measured with a caliper. Disc volume = (long diameter + short diameter)/2)^2^ × *π* × disc height. The surfaces of EPs were cleaned using a Pulsavac Wound Debridement Irrigation System (Zimmer, Minneapolis, USA) with Ringer's buffer to remove the cutting debris and blood clots. After prewashing in PBS with 10% P/S, IVDs were cultured in 6‐well plates with 7.5 mL IVD culture medium, DMEM supplemented with 1% P/S, 50 mg/mL Primocin (Invitrogen, San Diego, California), 2% FBS, 50 μg/mL ascorbate 2 phosphate, 1% ITS+, 1% NEAA, at 37°C, 5% CO_2_.

### 2.6 IVD culture and intradiscal injection

IVDs having a diameter of 1.5 to 2.0 cm were selected for the current study. IVDs were cultured free swelling during the night. Dynamic loading was performed, at 0.02 to 0.2 MPa, 0.2 Hz for 2 hours per day within a bioreactor.^7^ IVDs from each donor were randomly divided into three groups: PBS, TNF‐α and TNF‐α + Etanercept. TNF‐α + Etanercept: 40 μL of TNF‐α, containing 100 ng TNF‐α/cm^3^ of disc volume, was firstly injected into the disc, 30 minutes after 20 μL Etanercept, containing 10 μg Etanercept per 100 ng TNF‐α, was injected into the disc. TNF‐α: 40 μL of TNF‐α, containing 100 ng TNF‐α/cm^3^ of disc volume, was injected into disc 30 minutes after 20 μL PBS was injected. PBS: 40 and 20 μL PBS was injected into disc sequentially. The injection was performed using a 30‐gauge insulin needle, after the first dynamic loading on day 1. The intradiscal injection dose of Etanercept was kept at the same ratio of TNFα to Etanercept as in vitro, which is 1:100. IVDs were cultured with daily dynamic loading and free swelling recovery overnight, the disc size of IVDs was measured before and after loading.^7^ Culture media were collected daily after free swelling for further analysis. The NP tissue (gel‐like inner core of 6‐8 mm of diameter) was collected for gene expression analysis on day 2 and day 5.

### 2.7 Gene expression analysis

RNA samples were collected from monolayer NP cells by adding 0.5 mL TRI reagent (Molecular Research Centre Inc., Cincinnati, Ohio) with 2.5 μL polyacryl carrier (Molecular Research Centre Inc) per well. RNA isolation was performed according to the manufacturer's specifications. RNA isolation from NP tissues was performed as described before.^59^ NP tissues, 150 to 200 mg per sample isolated from discs, were cut into small pieces, snap‐frozen in liquid nitrogen and pulverized. The pulverized tissue was carefully collected and put into 3 mL TRI reagent with 15 μL polyacryl carrier. The volume of the TRI reagent was added according to the original NP tissue weight (3 mL TRI for 150‐200 mg tissue) with a volume ratio of >10:1 to supply adequate TRI volume for RNA isolation. Samples were homogenized immediately by a tissue‐lyser. After centrifugation, the supernatant was collected. Phase separation was performed by adding 100 μL bromochloropropane per 1 mL of TRI reagent and centrifugation. The aqueous phase was mixed with the same volume of 70% ethanol. The following steps were performed using the QIAGEN RNeasy MINI kit according to the manufacturer's protocol.

SuperScript VILO cDNA Synthesis Kit (Invitrogen) was used for cDNA synthesis with 400 ng RNA per sample. The quantitative real‐time polymerase chain reaction (qRT‐PCR) was conducted on QuantStudio6 PCR System (Applied Biosystems). The primers and probes used in qRT‐PCR for human and bovine samples are shown in Table 1. All the data were analyzed using 2^−ΔΔCT^ method, with RPLP0 as an endogenous control. The RPLP0 showed similar Ct value with different treatments, indicating TNFα and Etanercept did not show an influence on the house keeping gene expression.

**TABLE 1:** Oligonucleotide primers and probes (bovine and human) used for qRT‐PCR

Gene	Primer/probe type	Sequence
bIL6	Primer fw (5′‐3′)	TTC CAA AAA TGG AGG AAA AGG A
	Primer rev (5′‐3′)	TCC AGA AGA CCA GCA GTG GTT
	Probe (5′FAM/3′TAMRA)	CTT CCA ATC TGG GTT CAA TCA GGC GATT
bCOL2A1	Primer fw (5′‐3′)	AAG AAA CAC ATC TGG TTT GGA GAA A
	Primer rev (5′‐3′)	TGG GAG CCA GGT TGT CAT C
	Probe (5′FAM/3′TAMRA)	CAA CGG TGG CTT CCA CTT CAG CTA TGG
bACAN	Primer fw (5′‐3′)	CCA ACG AAA CCT ATG ACG TGT ACT
	Primer rev (5′–3′)	GCA CTC GTT GGC TGC CTC
	Probe (5′FAM/3′TAMRA)	ATG TTG CAT AGA AGA CCT CGC CCT CCA T
bMMP3	Primer fw (5′–3′)	GGC TGC AAG GGA CAA GGA A
	Primer rev (5′–3′)	CAA ACT GTT TCG TAT CCT TTG CAA
	Probe (5′FAM/3′TAMRA)	CAC CAT GGA GCT TGT TCA GCA ATA TCT AGA AAA C
bADAMTS5	Primer fw (5′–3′)	GAT GGT CAC GGT AAC TGT TTG CT
	Primer rev (5′–3′)	GCC GGG ACA CAC CGA GTA C
	Probe (5′FAM/3′TAMRA)	AGG CCA GAC CTA CGA TGC CAG CC
bADAMTS4	Primer fw (5′–3′)	CCC CAT GTG CAA CGT CAA G
	Primer rev (5′–3′)	AGT CTC CAC AAA TCT GCT CAG TGA
	Probe (5′FAM/3′TAMRA)	AGC CCC CGA AGG GCT AAG CGC
bCOX2		Bt03214492_m1
bIL8		Bt03211906_m1
bRPLP0		Bt03218086_m1
hACAN	Primer fw (5′–3′)	AGT CCT CAA GCC TCC TGT ACT CA
	Primer rev (5′–3′)	CGG GAA GTG GCG GTA ACA
	Probe (5′FAM/3′TAMRA)	CCG GAA TGG AAA CGT GAA TCA GAA TCA ACT
hMMP3		Hs00968305_m1
hIL8		Hs00174103_m1
hRPLP0	Primer fw (5′–3′)	TGG GCA AGA ACA CCA TGA TG
	Primer rev (5′–3′)	CGG ATA TGA GGC AGC AGT TTC
	Probe (5′FAM/3′TAMRA)	AGG GCA CCT GGA AAA CAA CCC AGC

*Note*: Primers and probes with the sequence shown were custom‐designed; primers and probes with the catalog number were from Applied Biosystems.

Abbreviations: ACAN, aggrecan; ADAMTS4, a disintegrin and metalloproteinase with thrombospondin motifs 4; ADAMTS5, a disintegrin and metalloproteinase with thrombospondin motifs 5; COL2A1, type II collagen; FAM, carboxyfluorescein; fw: forward; Gene prefix “b” bovine, prefix “h” human; rev, reverse; IL6, interleukin 6; IL8, interleukin 8; MMP3, matrix metalloproteinase‐3; RPLP0: Ribosomal Protein Lateral Stalk Subunit P0; TAMRA, tetramethylrhodamine.

### 2.8 Enzyme‐linked immunosorbent assay

IL‐6 and IL‐8 content in bovine IVD organ culture media were measured with enzyme‐linked immunosorbent assay (ELISA) kits (Kingfisher Biotech, St. Paul, Minnesota). Capture antibody: anti‐bovine IL‐6 polyclonal antibody (KP0652B‐100, Kingfisher Biotech), anti‐bovine IL‐8 polyclonal antibody (PB1164B‐100, Kingfisher Biotech). Detection antibody: Biotinylated‐anti‐bovine IL‐6 (KPB0653B‐050, Kingfisher Biotech), Biotinylated‐anti‐bovine IL‐8 (PBB1165B‐050, Kingfisher Biotech). Experiments were performed according to the manufacturer's protocol. The results of the ELISA were presented as the original concentration in the media without normalization.

### 2.9 Glycosaminoglycan and nitric oxide measurement

The amount of Glycosaminoglycans (GAGs) released in IVDs culture media was measured by using the 1,9‐dimethylmethylene blue dye method.^60^ The level of GAG release from each IVD at each time point after injection was normalized to the amount released on day 1 before injection by dividing the corresponding day's GAG release content with the amount of GAG release on day 1. The concentration of nitric oxide (NO) in the culture media of IVDs was detected as the level of its stable oxidation product, nitrite (NO^2−^), using the Griess Reagent Kit (Promega, USA). The NO concentrations in the media are presented in the results section without normalization.

### 2.10 Statistical analysis

Statistical analyses were performed using the GraphPad Prism 7 software (GraphPad Software, Inc., La Jolla, California). D'Agostino‐Pearson omnibus normality test was used to define whether the data were normally distributed. For data that were normally distributed, unpaired *t*‐test was used to determine differences between two groups; One‐way ANOVA was used to determine differences between three or more groups. For the not normally distributed data, Mann‐Whitney *U* test was used to determine differences between two groups; Kruskal‐Wallis test was used to determine differences between three or more groups. *P* < .05 was considered statistically significant.

## 3 RESULTS

### 3.1 Bovine and human recombinant TNF‐α comprise equivalent proinflammatory potency in bovine NP cells

**FIGURE 1:**
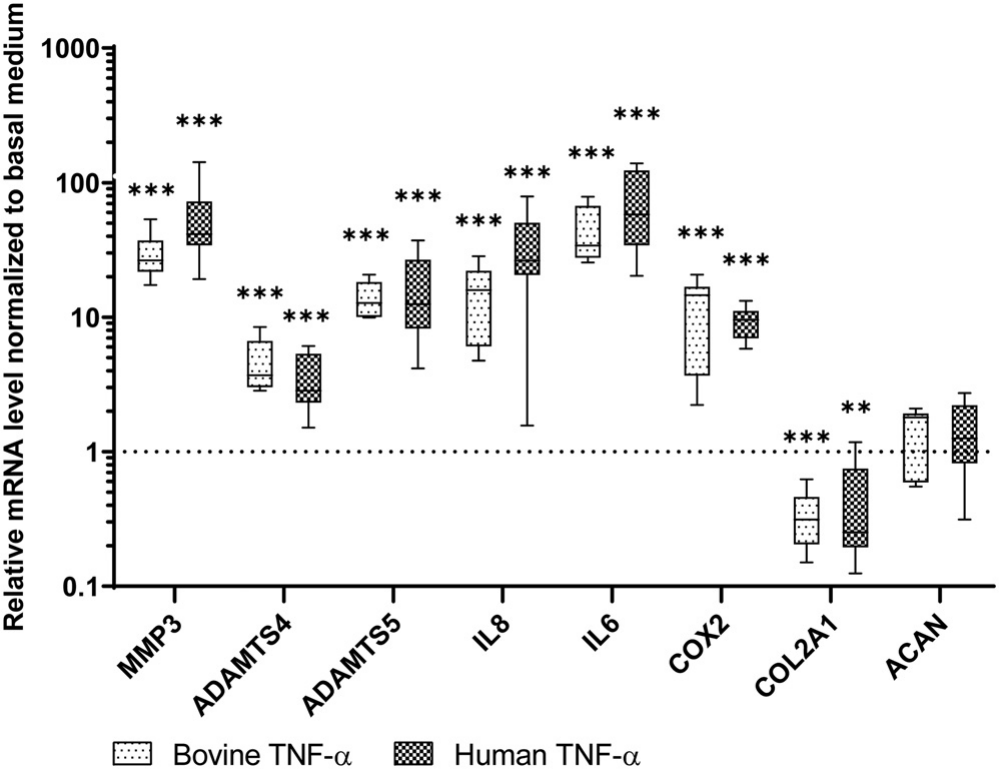
Relative mRNA expression level of bovine NP cells cultured with bovine or human recombinant TNF‐α. Bovine NP cells cultured with 10 ng/mL bovine or human recombinant TNF‐α for 72 hours. The mRNA expression level was normalized to the control group with basal medium. Min to Max with median and interquartile range, n = 9, **P < .01, ***P < .001 vs Basal Medium group

Bovine NP cells were treated with 10 ng/mL bovine or human recombinant TNF‐α. Catabolic gene expression as well as proinflammatory mediators are illustrated in Figure 1 (median and interquartile range). COL2A1 (0.31 (0.21 to 0.46) bovine, 0.25 (0.20 to 0.74) human) expression was significantly downregulated, while ACAN (1.80 (0.60 to 1.91) bovine, 1.26 (0.83 to 2.19) human) expression was not changed. Degradative proteinases, MMP3 (26.48 (21.98 to 36.76) bovine, 41.48 (34.61 to 71.81) human), ADATMS4 (3.70 (3.05 to 6.70) bovine, 2.84 (2.34 to 5.30) human), ADAMTS5 (12.78 (10.17 to 18.02) bovine, 12.50 (8.33 to 26.50) human), and inflammatory mediators, IL‐6 (34.13 (28.00 to 66.33) bovine, 57.99 (34.60 to 121.90) human), IL‐8 (15.90 (6.14 to 21.89) bovine, 26.32 (20.81 to 49.62) human), COX2 (14.55 (3.73 to 16.64) bovine, 9.54 (7.04 to 11.03) human), were significantly upregulated by both types of TNF‐α. There was no difference in the gene expression between treatment with human or bovine TNF‐α.

### 3.2 TNF‐α induced inflammation in human NP cells

**FIGURE 2:**
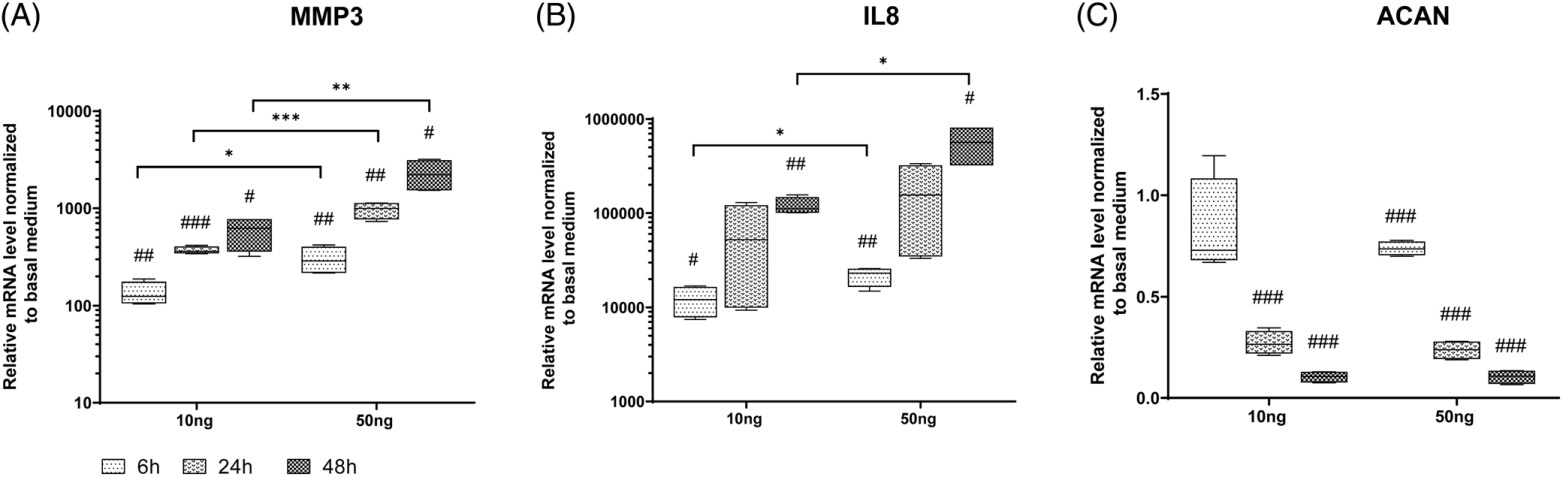
Relative mRNA expression of human NP cells treated with different dose of TNF‐α at different time points. Human NP cells treated with 10 ng/mL or 50 ng/mL TNF‐α for 6, 24, and 48 hours. Gene expression data of MMP3 (A), IL‐8 (B), ACAN (C) were normalized to the basal medium without TNF‐α as 1. Min to Max with median and interquartile range, n = 4, #*P* < .05, ##*P* < .01, ###*P* < .001 vs basal medium, *P < .05, **P < .01, ***P < .001, TNF‐α 10 ng/mL vs 50 ng/mL at the same time point

Human NP cells were treated with human recombinant TNF‐α at a concentration of 10 or 50 ng/mL. Samples were collected at three time points, 6, 24, and 48 hours, for gene expression analysis. As shown in Figure 2 (median and interquartile range, 10 ng: 6, 24, and 48 hours, 50 ng: 6, 24, and 48 hours respectively), MMP3 (124.7 (106.6‐175), 364.0 (348.4‐403.2), 624.3 (363.5‐765.0), 288(219.1‐402.5), 997.1 (775.3‐1123.0), 2222.0 (1544.0‐3104.0)) and IL‐8 (12 139 (7916‐16 423), 52 191(10 034‐120 284), 111 110 (101 742‐146 623), 23 167 (16 714‐25 564), 155 345 (35 201‐319 280), and 564 964 (324 997‐804 055)) expression were upregulated over time with a dose‐dependent effect. ACAN (0.73 (0.68‐1.08), 0.27 (0.22‐0.33), 0.11 (0.79‐0.13), 0.74 (0.71‐0.77), 0.24 (0.19‐0.28), and 0.11 (0.07‐0.13)) was downregulated over time independent of the TNF‐α dose.

**FIGURE 3:**
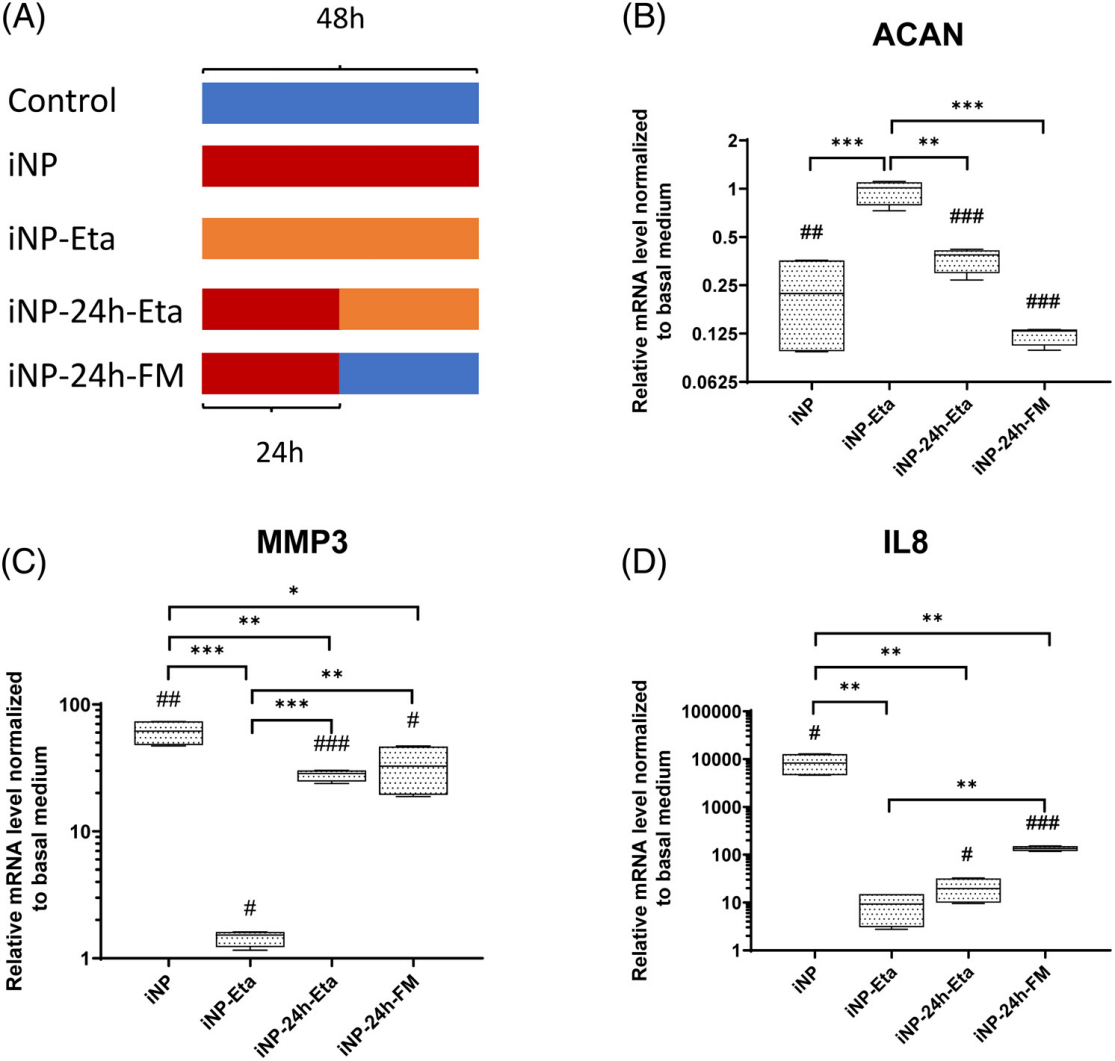
Relative mRNA expression levels of human NP cells. Human NP cells were cultured with basal medium (Control), TNF‐α 10 ng/mL for 48 hours (iNP), TNF‐α immediately followed by 1 μg/mL Etanercept for 48 hours (iNP‐Eta), TNF‐α for 24 hours followed by 1 μg/mL Etanercept added for 24 hours (iNP‐24 hours‐Eta), or TNF‐α for 24 hours followed by fresh basal medium without TNF‐α for 24 hours (iNP‐24 hours‐FM). (A) Scheme of treatment, blue: TNF‐α free, red: TNF‐α exist, orange: both TNF‐α and Etanercept exist. Gene expression data of ACAN (B), MMP3 (C), and IL‐8 (D) were normalized to the level of control group as 1. Min to Max with median and interquartile range, n = 4, #*P* < .05, ##*P* < .01, and ###*P* < .001 vs basal medium, *P < .05, **P < .01, and ***P < .001

To investigate the anti‐inflammatory treatment with TNF‐α blocking at different time points, NP cells were treated with TNF‐α 10 ng/mL for 48 hours (iNP), TNF‐α immediately followed by 1 μg/mL Etanercept for 48 hours (iNP‐Eta), TNF‐α for 24 hours followed by 1 μg/mL Etanercept for 24 hours (iNP‐24 hours‐Eta), and finally TNF‐α for 24 hours then replacing to fresh basal medium without TNF‐α for 24 hours (iNP‐24 hours‐FM). NP cells treated with basal medium served as control (Figure 3; median and interquartile range). Inflammation induced by TNF‐α (iNP) caused an increased MMP3 (61.7 (48.3‐73.1)) and IL‐8 (8289 (4700‐12 572)) expression and decreased ACAN (0.22 (0.10‐0.35)) expression. Etanercept applied at the beginning of the proinflammatory processes completely inhibited inflammation in iNP‐Eta, as shown by decreased MMP3 (1.5 (1.2‐1.6)) and IL‐8 (9.3 (3.1‐14.7)) expression and increased ACAN (1.01 (0.80‐1.09)) expression compared with iNP group, but comparable to the control group. Etanercept treatment in the middle of the inflammation process can block the inflammation effect, as shown by decreased IL‐8 (19.6 (10.1‐31.4)) expression and partly decreased MMP3 (28.6 (24.9‐30.0) compared with iNP. However, the ACAN (0.39 (0.30‐0.41) expression was comparable with iNP. Removal of TNF‐α after 24 hours (iNP‐24 hours‐FM) showed the similar effect as iNP‐24 hours‐Eta, partial recovery from inflammation, observed by partly decreased IL‐8 (135.6 (121.3‐149.6) and MMP3 (32.64 (19.51‐46.40) expression compared with iNP, but ACAN (0.13 (0.11‐0.13) expression cannot be recovered.

### 3.3 Proinflammatory IVD organ culture model

According to our previous study, intradiscal injection of 100 ng human TNF‐α per disc did not induce a significant inflammatory effect.^7^ As shown in Figure 1, both bovine and human TNF‐α can induce inflammation equally in bovine NP cells. Hence, the difference in species origin of TNF‐α was excluded. Therefore, we hypothesized that the dose of TNF‐α may influence the results. A preliminary experiment was performed by intradiscal injection of 100, 200, or 400 ng human recombinant TNF‐α into IVDs with various sizes (1.5‐3 cm^3^). Results (Figure S1) showed a trend of enhanced disc inflammation and its response with increasing TNF‐α dose, evaluated by NO and GAG release in IVD culture media. When results were normalized to the injected TNF‐α amount/cm^3^ disc volume, a threshold at 100 ng TNF‐α/cm^3^ disc volume was observed, with significant inflammatory effect above this injection dose.

**FIGURE 4:**
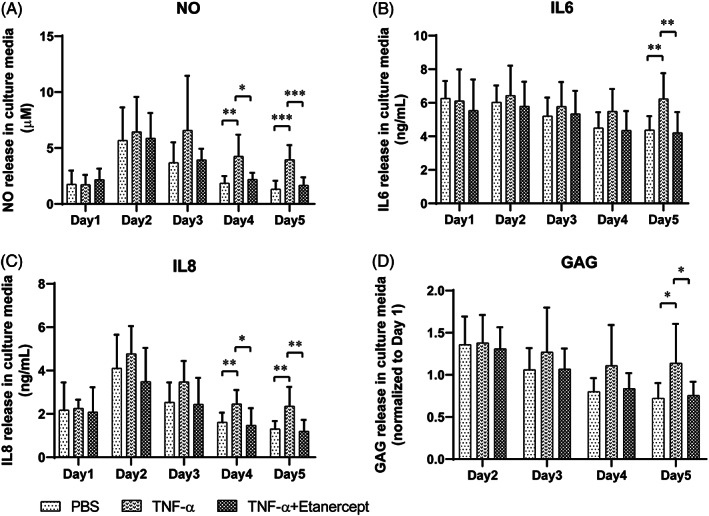
NO, IL‐6, IL‐8, and GAG release in the IVD culture medium. NO (A), IL‐6 (B), IL‐8 (C), and relative GAG (D, normalized to day 1) release in the conditioned medium of IVDs with PBS injection (PBS), TNF‐α injection (TNF‐α), and TNF‐α plus Etanercept injection (TNF‐α + Etanercept). Intradiscal injection performed after day 1 loading. Mean + SD, n = 9, *P < .05, **P < .01, and ***P < .001

**FIGURE 5:**
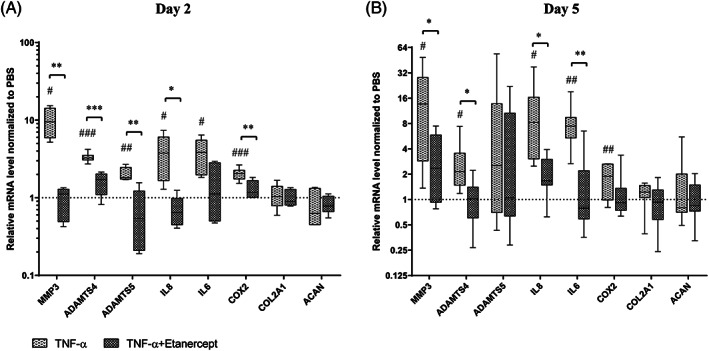
Relative mRNA expression level of NP tissue from IVDs. IVDs cultured with TNF‐α injection (TNF‐α), and TNF‐α plus Etanercept injection (TNF‐α + Etanercept), the gene expression level in NP tissue on day 2 (A) and day 5 (B), data were normalized to IVDs with PBS injection (PBS) as 1. Min to Max with median and interquartile range, samples on day 2, n = 5, samples on day 5, n = 8, #*P* < .05, ##*P* < .01, and ###*P* < .001 vs PBS group, *P < .05, **P < .01, and ***P < .001, TNF‐α group vs TNF‐α + Etanercept group

**FIGURE 6:**
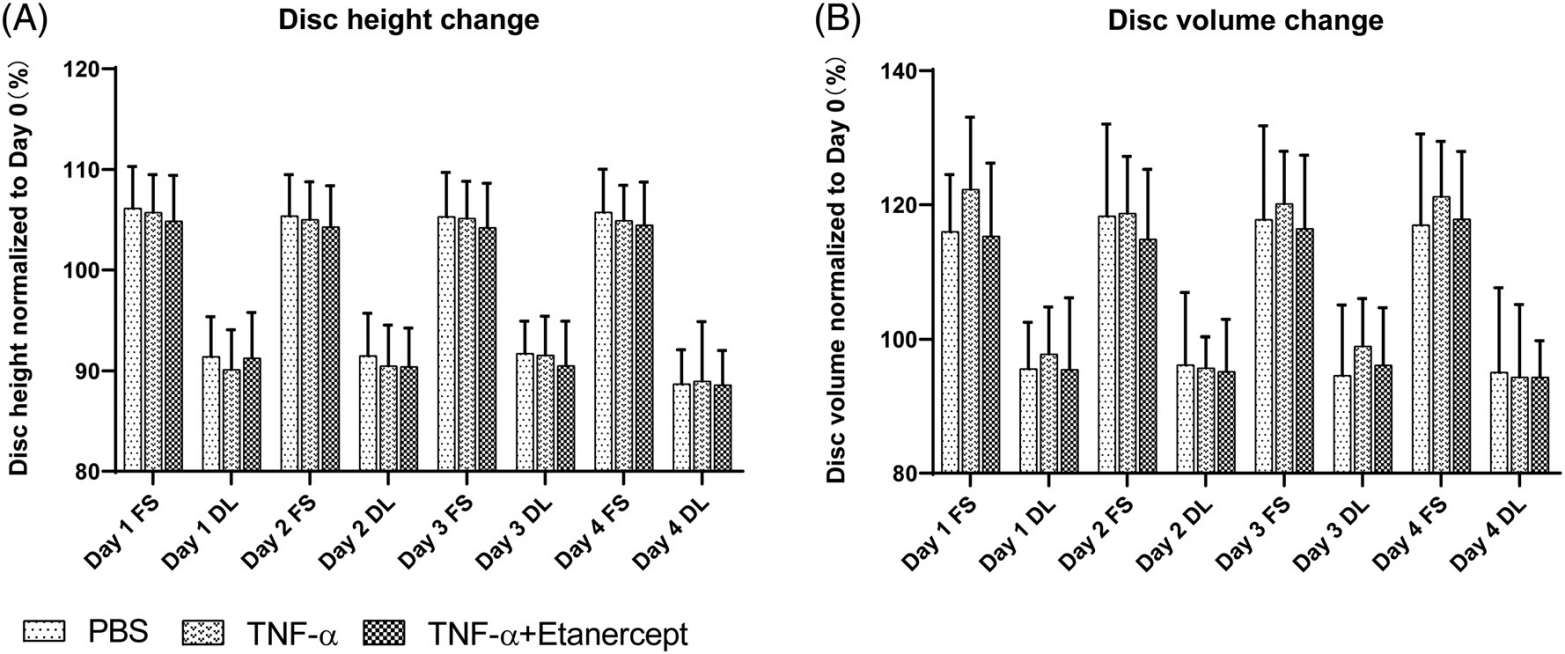
Disc height and volume change of cultured IVDs. IVDs with PBS injection (PBS), TNF‐α injection (TNF‐α), and TNF‐α plus Etanercept injection (TNF‐α + Etanercept) at different time points: after free swelling culture overnight (FS) and after dynamic loading (DL) over 5 days of organ culture. Data were normalized to initial disc height (A) or volume (B) after dissection on Day 0 in percentage. Mean + SD, n = 9

Consequently, intradiscal injection was performed with TNF‐α at 100 ng/cm^3^ disc volume after the first dynamic loading on day 1 to induce inflammation in the IVD organ culture model (TNF‐α). Etanercept was injected 30 minutes after TNF‐α injection, at a ratio of Etanercept(w): TNF‐α(w) = 100:1 as anti‐inflammation positive control (TNF‐α + Etanercept). Discs were injected with the same volume of PBS as negative control (PBS). Discs were cultured with daily physiological loading and culture media after overnight free swelling were collected daily for NO, IL‐6, IL‐8, and GAG measurement. NP tissue was collected at two time points, at 1 (day 2) or 4 days (day 5) after TNF‐α injection. As shown in Figure 4 (Mean ± SD), starting from day 4, TNF‐α injected discs released significantly higher NO (5.73 ± 5.02) and IL‐8 (2.20 ± 0.78) compared with PBS (2.08 ± 0.91 NO, 1.61 ± 0.44 IL‐8) and TNF‐α + Etanercept (2.70 ± 1.61 NO, 1.60 ± 0.84 IL‐8). On day 5, TNF‐α injected discs showed significantly higher amount of GAG (1.34 ± 0.47) and IL‐6 (6.24 ± 1.53) release compared with PBS (0.72 ± 0.18 GAG, 4.38 ± 0.82 IL‐6) and TNF‐α + Etanercept (0.76 ± 0.16 GAG, 4.21 ± 1.23 IL‐6). As shown in Figure 5 (median and interquartile range, day 2: TNF‐α, TNF‐α + Etanercept, day 5: TNF‐α, TNF‐α + Etanercept, respectively), the gene expression of MMP3 (9.59 (5.90‐14.23), 1.12 (0.49‐1.29), 9.22 (1.23‐19.94), 2.35 (0.92‐5.85)), ADAMTS4 (3.25 (3.06‐3.51), 1.70 (1.09‐2.03), 2.14 (1.48‐3.56), 1.02 (0.60‐1.41)), IL‐8 (3.76 (1.66‐6.07), 0.65 (0.45‐0.99), 5.17 (2.07‐14.16), 1.67 (1.48‐3.01)), IL‐6 (3.85 (1.96‐5.51), 1.12 (0.50‐2.82), 7.43 (3.19‐8.94), 0.79(0.59‐2.20)), and COX2 (2.06 (1.74‐2.27), 1.18 (1.02‐1.67), 1.89 (0.98‐2.64), 0.92 (0.74‐1.36)) were significantly increased at day 2 and day 5 by TNF‐α injection, and ADAMTS5 (1.81(1.73‐2.47), 0.54 (0.21‐1.22), 2.53 (0.70‐13.78), and 1.05 (0.63‐10.66)) was upregulated at day 2. All genes' upregulation can be eliminated by Etanercept. Nevertheless, COL2A1 (1.03 (0.79‐1.40), 0.90 (0.80‐1.28), 1.22 (1.05‐1.47), 0.93 (0.58‐1.30)), and ACAN (0.63 (0.45‐1.32), 0.78 (0.66‐1.05), 0.80 (0.70‐2.02), 0.84 (0.72‐1.49)) expression were not changed by TNF‐α. After free swelling disc height increased by proximately 5% and disc volume increased by proximately 18%. After daily loading disc height decreased by approximately 10% and disc volume by approximately 5%, compared with day 0 when discs were isolated. However, the fold changes of disc height and volume did not show any difference among these three groups (Figure 6).

## 4 DISCUSSION

Anti‐inflammatory therapy has been considered as a promising approach to delay the IVD degeneration and relieve discogenic pain. TNF‐α, as a pro‐inflammatory factor, has been reported to be associated with IVD degeneration and discogenic pain.^21^, ^61^ Anti‐inflammatory therapies targeting TNF‐α are widely reported, with preserved matrix production and restraint of matrix degradation.^31^, ^37^, ^42^ Therefore, in vitro and ex vivo IVD inflammatory culture systems induced by TNF‐α are clinically relevant models for drug development for treatment of disc degeneration.

In the current study, several specific questions related to the inflammatory model were investigated. Firstly, due to the scarce access to human IVD tissue and especially to healthy samples, bovine IVD cells and bovine caudal whole IVDs have been widely used in spine research. While using TNF‐α for inflammation induction of bovine disc cells or organs, one question which has not been well addressed is whether human and bovine recombinant TNF‐α imply the same effect on bovine disc cells, or whether the TNF‐α receptors on bovine disc cells can also transmit the signaling from human recombinant TNF‐α. Our results showed both bovine and human recombinant TNF‐α can equally induce inflammation in bovine NP cells in vitro (Figure 1). These results support most of the studies in the field, confirming that human recombinant TNF‐α can be used for inflammation induction in bovine disc cells.^38^, ^62^

Secondly, the required dose for an inflammatory IVD organ culture model based on intradiscal TNF‐α injection is unclear. Takahashi et al reported that the concentration of TNFa in herniated disc tissue is a dozens of pgs per 100 mg tissue.^32^ However, the dose of TNF‐α used in all the artificial inflammatory models is much higher than the pathological dose, since such low dose of TNF‐α may fail to induce significant inflammation response or need a very long time to reach significance in vitro and ex vivo. According to our previous study, TNF‐α injection at a fixed dose of 100 ng per disc did not induce a consistent inflammatory response.^7^ In contrast, TNF‐α added into disc culture media at a dose 200 ng/mL induced significant inflammation in discs without cartilage endplates.^38^ The current study showed that an injection dose normalized to the disc volume was necessary to induce a reproducible inflammatory effect in the IVD organ culture model. The injection dose was optimized to 100 ng TNF‐α/cm^3^ disc volume. This has effectively induced inflammation in bovine NP tissue, as shown by increased NO, GAG, IL‐6, and IL‐8 release in culture media, and upregulated MMP3, ADAMTS4, IL‐8, IL‐6, and COX2 expression in NP tissue on both day 2 and day 5. These results showed that TNF‐α intradiscal injection at the adjusted dose increased expression of catabolic enzymes and inflammatory mediators in the whole IVD organ culture system, which is consistent with previous NP tissue and cell culture studies.^34^, ^45^ This may be a realistic way to mimic the inflammatory and degenerative condition of IVD disease in preclinical models.

Our result showed that TNF‐α downregulated gene expression of type II collagen in cell culture, but not in whole organ culture. In contrast, Seguin et al showed that TNF‐α decreased expression of type II collagen in NP tissue culture.^34^ This suggests that the whole IVD organ culture system is beneficial for maintaining disc cell homeostasis, which may be due to the physiological osmolarity inside the intact organ that has been shown to maintain the NP tissue specific matrix composition.^51^

Annular puncture may induce disc degeneration depending on the disc size and needle size.^63^ A recent goat study revealed that 22G needle puncture did not result in degenerative changes in lumbar IVDs, nor was degeneration found in IVDs of Beagles injected using 25G needles.^64^, ^65^ Also in bovine caudal IVD we found that IVD puncture using a 30‐gauge needle did not cause dysregulation on expression of anabolic, catabolic and inflammatory markers.^7^ Therefore, injection using a 30‐gauge needle is not expected to cause an effect on the state of IVD degeneration in the current experiments.

Analysis of the culture medium was undertaken to investigate whether the molecule release was related to the disc volume. This was performed with IVDs cultured under physiological loading and without TNF‐α injection. The initial GAG release on day 1 from discs of different donors showed a high variation, which may result in an inundating difference between experimental groups. The day 2 GAG release was highly related with day 1, evaluated with linear regression (*R* ^2^ = .935, Figure S2). The NO, IL‐6, and IL‐8 release data did not show such inter‐donor variation (Figure S2). Therefore, the results of GAG release from the inflammatory model experiments (Figure 4D) were analyzed with normalized relative fold changes instead of using the original absolute content.

TNF‐α induced a nonrecoverable catabolic shift of NP cells even when it was removed from the medium at 24 hours after supplementation, which is consistent with previous studies.^38^, ^42^ More interestingly, our results showed that the time point of anti‐inflammatory treatment with Etanercept is crucial for reversing the catabolic effect caused by TNF‐α, where only Etanercept application at early time point could show a positive effect. This may explain the available clinical data where intradiscal Etanercept injection in patients with back pain showed controversy in pain relieving results. Etanercept epidural injection in patients with lumbosacral radicular pain of 6 to 26 weeks duration provided clinically significant reductions in mean daily worst leg pain and worst back pain.^66^ However, Etanercept injection in patients with chronic LBP, more than 6 months' duration, was unable to resolve chronic discogenic pain.^67^ Hence, anti‐inflammatory treatment with Etanercept at early onset of disc inflammation may be beneficial to relieve discogenic pain by reversing the degenerative cascade. There seems to be a time‐point dependent window of therapeutic applicability for anti‐inflammation strategies. However, radicular pain indicates IVD herniation, which is a different entity from chronic LBP related to IVD degeneration and may therefore intrinsically respond differently to anti‐inflammatory treatment. In clinics, patients are usually treated at a certain period after an acute inflammation or during chronic inflammation process. At this stage, targeting or removal of the inflammatory factor may not be sufficient. Also, treatment to prevent continuous degeneration needs to be included as well.

Limitations: This study solely focused on TNF‐α induced acute inflammation within IVDs. Other proinflammatory factors such as IL‐1β and lipopolysaccharide may also be used for the same purpose, while the differences in the effects of various factors need to be further evaluated. Both in vitro and ex vivo experiments were only performed within 1 week. Therefore, further studies should be designed to investigate the effect of prolonged or repeated stimulation of TNF‐α. The exogenous dose of TNF‐α in the current study is much higher than in vivo pathological conditions, and a high dose of TNF‐α can induce cell apoptosis and senescenc, which play important roles in IVD degeneration..^32^, ^38^, ^68^ In rat NP cells cultured with TNF‐α at 50 ng/mL for 12 hours apoptosis was induced.^68^ Also in IVD organs cultured with 200 ng/mL TNF‐α for 21 days cell senescence was induced.^38^ Further study is warranted to evaluate the effect of TNF‐α on cell apoptosis and senescence in long‐term within the current model in the future.

## 5 CONCLUSION

The present work sought to address several specific questions on the establishment of an IVD inflammatory model with TNF‐α. Bovine and human recombinant TNF‐α induced equal inflammatory effects in bovine NP cells. A bovine whole IVD inflammatory model was established by intradiscal injection of 100 ng TNF‐α/cm^3^ disc volume, as indicated by increased NO, GAG, IL‐6, IL‐8 release in culture media, and upregulated MMP3, ADAMTS4, IL‐8, IL‐6, and COX2 expression in NP tissue. The time points of anti‐inflammatory treatment are crucial, and additional anti‐catabolic treatment to prevent degeneration would be needed to completely maintain disc biology and function.

## CONFLICT OF INTEREST

The authors have no conflict of interest.

## AUTHOR CONTRIBUTIONS

Jie Du: substantial contributions to study design, acquisition, analysis, interpretation of data, drafting the article, revising it critically, and final approval. Judith‐J. Pfannkuche: substantial contributions to acquisition of data, analysis, interpretation of data, revising the article critically, and final approval. Gernot Lang, Sonja Häckel, Laura B. Creemers, Mauro Alini, and Sibylle Grad: substantial contributions to study design, revising the article critically, and final approval. Zhen Li: substantial contributions to study design, interpretation of data, drafting the article, revising it critically, final approval, and takes responsibility for the integrity of the work as a whole, from inception to finished article.

## Supporting information


**Figure S1** Preliminary experiments for optimization of the dose of intradiscal injection of TNF‐α. Eight discs obtained from two tails, four discs per tail. After day 1 loading, discs from each tail were randomly injected with PBS, 100, 200, or 400 ng TNF‐α respectively. The dose of TNF‐α in each disc was calculated as a concentration in ng per cm^3^ disc volume. Discs were cultured with daily physiological loading over 5 days, GAG (A) and NO (B) release in culture media after overnight free swelling were measured. Each bar represent the result of one disc. No statistical analyze was applied.

**Figure S2** Regression analysis of the GAG, NO, IL‐6, and IL‐8 release content in the conditioned media of bovine IVDs cultured during day 1 and day 2, under physiological culture condition without TNF‐α injection.
