# Supplementary material for: Proinflammatory intervertebral disc cell and organ culture models induced by tumor necrosis factor alpha
Source: JOR Spine. 2020 Jun 19;3(3):e1104. doi: 10.1002/jsp2.1104 (PMC7524256; doi:10.1002/jsp2.1104)
Supplement: Supplementary file 1 — Figure S1 Preliminary experiments for optimization of the dose of intradiscal injection of TNF‐α. Eight discs obtained from two tails, four discs per tail. After day 1 loading, discs from each tail were randomly injected with PBS, 100, 200, or 400 ng TNF‐α respectively. The dose of TNF‐α in each disc was calculated as a concentration in ng per cm3 disc volume. Discs were cultured with daily physiological loading over 5 days, GAG (A) and NO (B) release in culture media after overnight free swelling were measured. Each bar represent the result of one disc. No statistical analyze was applied. [file JSP2-3-e1104-s001.docx]

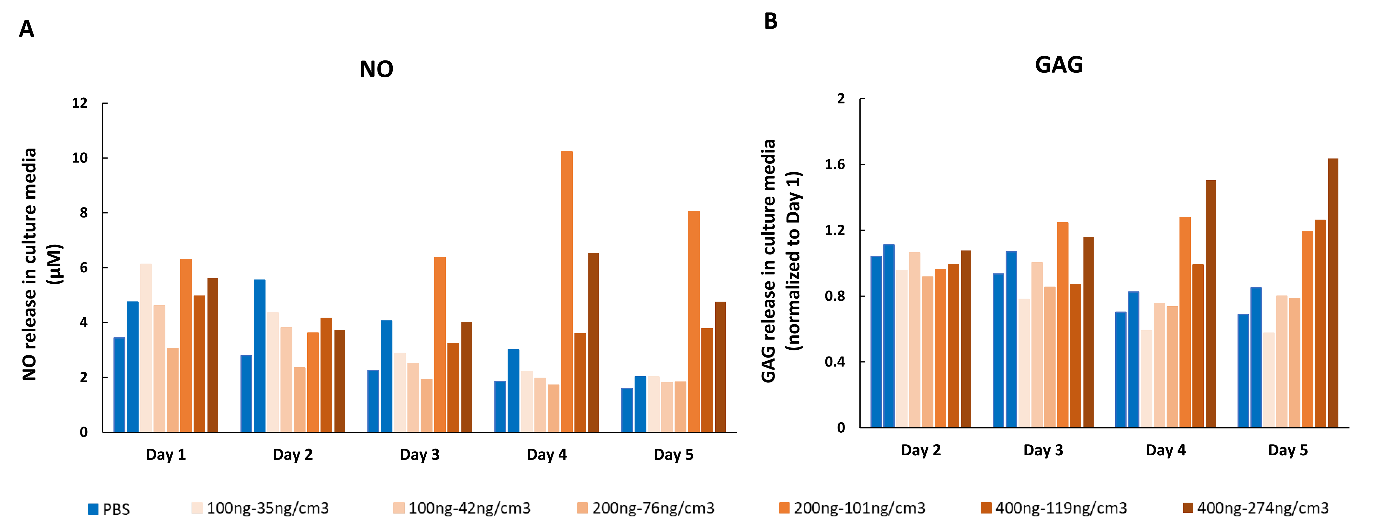


**Supplementary data figure 1.** Preliminary experiments for optimization of the dose of intradiscal injection of TNF-α. Eight discs obtained from 2 tails, 4 discs per tail. After day 1 loading, discs from each tail were randomly injected with PBS, 100 ng, 200 ng or 400 ng TNF-α respectively. The dose of TNF-α in each disc was calculated as a concentration in ng per cm^3^ disc volume. Discs were cultured with daily physiological loading over 5 days, GAG (A) and NO (B) release in culture media after overnight free swelling were measured. Each bar represent the result of one disc. No statistical analyse was applied.
