# Supplementary material for: Proinflammatory intervertebral disc cell and organ culture models induced by tumor necrosis factor alpha
Source: JOR Spine. 2020 Jun 19;3(3):e1104. doi: 10.1002/jsp2.1104 (PMC7524256; doi:10.1002/jsp2.1104)
Supplement: Supplementary file 2 — Figure S2 Regression analysis of the GAG, NO, IL‐6, and IL‐8 release content in the conditioned media of bovine IVDs cultured during day 1 and day 2, under physiological culture condition without TNF‐α injection. [file JSP2-3-e1104-s002.docx]

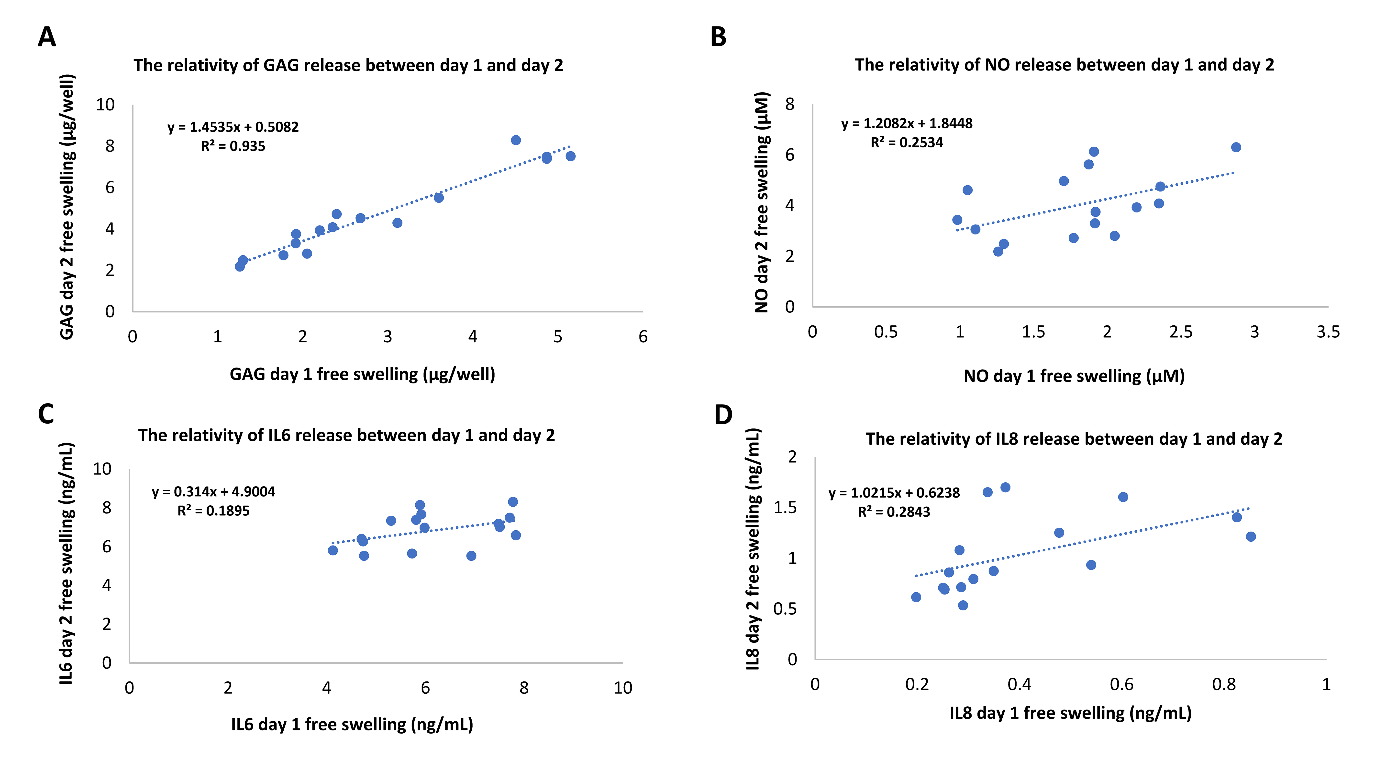


**Supplementary data figure 2.** Regression analysis of the GAG, NO, IL-6 and IL-8 release content in the conditioned media of bovine IVDs cultured during day 1 and day 2, under physiological culture condition without TNF-α injection.
